# Effects of Systemic Simvastatin on the Concentrations of Visfatin, Tumor Necrosis Factor-*α*, and Interleukin-6 in Gingival Crevicular Fluid in Patients with Type 2 Diabetes and Chronic Periodontitis

**DOI:** 10.1155/2018/8481735

**Published:** 2018-08-15

**Authors:** Maha A. Bahammam, Mai S. Attia

**Affiliations:** ^1^Department of Periodontology, Faculty of Dentistry, King Abdulaziz University, P.O. Box 80209 Jeddah 21589, Saudi Arabia; ^2^Department of Oral Medicine, Periodontology, and Oral Diagnosis, Faculty of Dental Medicine, Al-Azhar University, Cairo, Egypt

## Abstract

**Purpose:**

The objective of this study is to explore the relationship between the levels of interleukin- (IL-) 6, tumor necrosis factor- (TNF-) *α*, and visfatin and simvastatin usage, in the gingival crevicular fluids (GCFs) of diabetic patients afflicted with chronic periodontitis.

**Methods:**

Eighty outpatients at the Periodontology Department, Faculty of Dentistry, University Dental Hospital (King Abdulaziz University), were categorized into 4 groups (20 patients per group), on the basis of radiological evaluation of bone loss, clinical attachment levels (CAL), probing depth (PD), and gingival indices: group 1 (healthy periodontium), group 2 (chronic periodontitis + type 2 diabetes), group 3 (chronic periodontitis), and group 4 (type 2 diabetes + chronic periodontitis + simvastatin). Enzyme-linked immunosorbent assays were used to measure IL-6, TNF-*α*, and visfatin levels.

**Results:**

Significantly elevated levels of IL-6, TNF-*α*, and visfatin were seen in group 2 in comparison to groups 1 and 3. Reduced levels were seen in group 4 due to simvastatin usage. Positive association was seen between periodontal variables and the levels of IL-6, TNF-*α*, and visfatin.

**Conclusion:**

Periodontal destruction and diabetes have a synergistic effect on the elevation of inflammatory cytokine levels. Simvastatin may be beneficial in improving periodontal health among diabetic patients.

## 1. Introduction

Bone destruction, attachment loss, and gingival inflammation frequently present as the consequences of periodontal disease, which is both inflammatory and chronic in nature. There is a release of substances during immune responses; these interact with periodontopathogenic bacteria to result in the onset and progression of chronic periodontitis. The pathogenesis of periodontal disease may result due to a variety of genetic, environmental and systemic variables. Periodontal disease and diabetes mellitus (DM) have relatively high incidences in the general population with some degree of immune-regulatory dysfunction in their pathogenesis. DM is a metabolic condition that may have an impact on the occurrence and progression of periodontal diseases. This is supported by a few epidemiological and clinical studies, which indicate a higher incidence and intensity of periodontal diseases in patients afflicted with DM. Additionally, the occurrence of significant alveolar bone loss and periodontitis is 4.2-fold and 2.8-fold higher in type 2 diabetics in comparison to healthy individuals, respectively [1]. Additionally, an association between weakened periodontal health and inadequate glycemic regulation has been made, due to which periodontal diseases are regarded as the sixth complication of DM.

The greater incidence of debilitating periodontal disease in diabetics may be explained through a likely inflammatory mechanism. Specifically, the close relevance of the cytokine ‘tumor necrosis factor-*α*' (TNF-*α*) in DM and debilitating periodontal diseases has been observed. Both metabolic syndrome and obesity see a systemic elevation in the levels of TNF-*α* [2]. Animal and human model-based studies carried out in the 1990s were indicative of TNF-*α* secretions by adipocytes. The studies highlighted that these TNF-*α* secretions led to systemic chronic inflammation. Additionally, TNF-*α* induces resistance to insulin in both diabetic and obese patients. Conversely, the progress of debilitating periodontal diseases is associated with TNF-*α*, which acts as a primary and early periodontal pathogen-induced inflammatory cytokine in this context. Interleukin- (IL-) 6 is an additional cytokine that serves various functions. This is synthesized within the endothelium in addition to neutrophils and macrophages. However, there are complexities faced while exploring the significance of IL-6 under normal or disease conditions, due to the presence of both pro- and anti-inflammatory effects (double-edge effect). In the context of debilitating periodontal diseases, an elevation is seen in the levels of systemic IL-6 and gingival crevicular fluid (GCF) [3].

Adipocytes secrete visfatin, which is a human pre-B-cell colony enhancing factor in addition to being a 52 kDa molecule. While the infection and inflammation phases are ongoing, visfatin plays a suggested role in bringing about the synthesis of several cytokines; these are inclusive of IL-6, TNF-*α*, and IL-1*β*. The modulation of visfatin's expression is carried out in an analogous pattern such as that observed in the cytokine response to injury and infection [4]. Furthermore, periodontal disease progression might be evaluated by measuring the levels of various cytokines in systemically compromised patients. Moreover, simvastatin may have a role in the reduction of the inflammatory burden. Thus, the objective of this study is to explore the relationship between IL-6, TNF-*α*, and visfatin levels and simvastatin treatment through analyzing the GCFs of patients afflicted with chronic periodontitis and type 2 diabetes.

## 2. Methodology

### 2.1. Participants

This study incorporated the participation of every eligible outpatient at the Periodontology Clinic within the Faculty of Dentistry, University Dental Hospital at King Abdulaziz University, until the necessary sample quota for each category was fulfilled. Criteria for eligibility was inclusive of obtaining the agreement of these patients to participate in the research. In total, 80 participants were recruited, who consisted of 45 male participants and 35 female participants ranging in age between 32 and 50 years. These patients were divided equally into four categories of interest. Patients having a healthy periodontium were sorted into group 1. Group 2 incorporated diabetic patients who were additionally afflicted with chronic periodontitis; whereas, group 3 was composed of participants who were afflicted with chronic periodontitis only. Lastly, group 4 was composed of those diabetic patients suffering from chronic periodontitis who had undergone treatment using 20 mg simvastatin as a lipid-lowering agent between five and ten years.

Data was obtained from clinical examination and a direct interview questionnaire, which included topics on diabetes, condition of periodontitis, use of simvastatin, its dose, and how long the drug had been used. The exclusion criteria for this study encompassed patients afflicted with rheumatoid arthritis, tumors, heart diseases, gross oral pathology, smoking trends, hypertension, and aggressive periodontitis. Additionally, those patients were not included who took any kind of medicine or drugs and who were afflicted with additional systemic conditions which could potentially impact the progress of periodontal diseases. Furthermore, the exclusion criteria encompassed patients having undergone periodontal therapy in addition to patients having taken medicine with a potential impact on the status of periodontal diseases, within or 6 months prior to the conduction of this research. For ensuring a traumatic process of GCF collection, the use of microcapillary pipettes was incorporated. Using these pipettes further ensures the acquisition of undiluted GCF samples.

### 2.2. Ethical Considerations

The Institutional Ethical Committee and Review Board of the Faculty of Dentistry approved the ethics of this study. This faculty was present at the King Abdulaziz University in Jeddah, Saudi Arabia. Informed and written consent to voluntarily participate was received from every study participant and a clear explanation of the research was provided to them. Ethical approval was gained from King Abdulaziz University's Institutional Ethical Committee on Human Research. Furthermore, the stipulations put forth by the World Medical Association's Declaration of Helsinki and the CONSORT group were stringently followed with respect to carrying out and reporting the study [5].

### 2.3. Selection of Sites and Acquisition of GCFs

A periodontal probe (UNC-15; Hu-Friedy, Chicago, IL, USA) was used for exploring the clinical parameters. The first examiner (MS) carried out the group allocations, radiography, and selected sites for sampling purposes. In the succeeding day, sample collection was carried out by another examiner (MB). This step was necessary so that blood from the inflamed perioontia probing would not contaminate the GCF. In every group aside from group 1, a single site was chosen for sampling. Whereas, approximately 3 to 5 sites free from inflammation were chosen for every participant belonging to group 1, so that suitable quantities of GCF could be collected. Sampling was carried out for sites, which demonstrated the most inflammatory signs alongside bone loss, as validated through radiography. Additionally, sampling was conducted for sites representing the highest clinical attachment levels (CAL).

Periodontal charting was carried out for the full mouth of every patient. Parameters such as gingival index, probing depth (in mm), and CAL (in mm) were determined. The long-cone technique was used for taking periapical radiographs. These were then used for the evaluation of the related site with respect to bone density. An indirect digital image radiographic system and the DBSWIN software, which is part of the recently introduced Vista Scan system (Dürr Dental Bietigheim, Bissingen, Germany) were used to obtain and calculate pixel gray measurements (mineral content).

On the following day, removal of supragingival plaque was carried out in a way that the marginal gingiva was not touched. This was conducted after the chosen site was dried gently. After this step, the chosen region was isolated using cotton rolls, so that contamination due to saliva could be prevented. A microcapillary pipetted was positioned on the gingival sulcus's opening and the gingival margin was touched in a gentle manner; this ensured the collection of GCF. Volumetric microcapillary pipettes (Sigma-Aldrich, St. Louis, MO, USA) were used as calibration for collecting a 1 mL standardized volume; these pipettes were calibrated to 1.5 mL and colour-coded white.

Collecting every sample took place under ten minutes and exclusion of sites with no GCF expression was carried out within this time duration. This exclusion was necessary so that trauma caused to the site may be avoided. The study eschewed the use of micropipettes which were allegedly contaminated with saliva or blood. Prompt transfer of the collected samples into plastic vials was carried out. These vials were airtight and used for storing samples at −70°C until assaying was carried out.

### 2.4. Assay of GCF's Visfatin, IL-6, and TNF-*α* Levels

The levels of visfatin, IL-6, and TNF-*α* were ascertained through the use of an enzyme-linked immunosorbent assay (ELISA). As per the manufacturer's instructions, the use of the following kits was seen: AviBion Human IL-6 ELISA Kit (Cat number IL06001, Ani Biotech Oy, Orgenium Laboratories); RayBio Human Visfatin ELISA Kit (RayBiotech, Inc., Norcross, GA, USA) and AviBion Human TNF-*α* ELISA Kit (Cat number TNFa021; Ani Biotech Oy, Orgenium Laboratories, Vantaa, Finland). Each assay was conducted in duplicate. Thawing for every reagent was carried out prior to usage; specifically, these were maintained at room temperature or between 18°C and 25°C.

For each marker (100 *μ*L), the addition of anibodies within the relevant well in the kit was carried out. Incubation of these antibodies took place for approximately 1.5 hours. Following this, removal of the solutions took place. Furthermore, a wash solution was used for washing the wells four times. Furthermore, the removal of every solution was carried out and the wash solution was then used for washing the wells five times. Within every well, 100 *μ*L of a one-step substrate reagent (tetramethylbenzidine) was incorporated. Room temperature incubation was then carried out under dark conditions for a time duration of thirty minutes. Within every well, 50 *μ*L of stop solution was incorporated. An ELISA reader (Molecular Dynamics Inc., Sunnyvale, CA, USA) was used for measuring the 450 nm absorbance.

### 2.5. Statistics

The collection, revision, and statistical analysis of the data was carried out through the use of SPSS (version 20.0; IBM Corp., Armonk, NY, USA). One-way analysis of variance was used to compare the quantitative data in multiple groups (greater than 2). Following this, post hoc analysis was carried out. Additionally, the association between two variables of quantitative interest present within the same groups was carried out through the coefficient of Pearson correlation. Moreover, a *P* value less than 0.05 was chosen to represent statistically significant data.

## 3. Results

### 3.1. Biochemical Results

**Figure 1:**
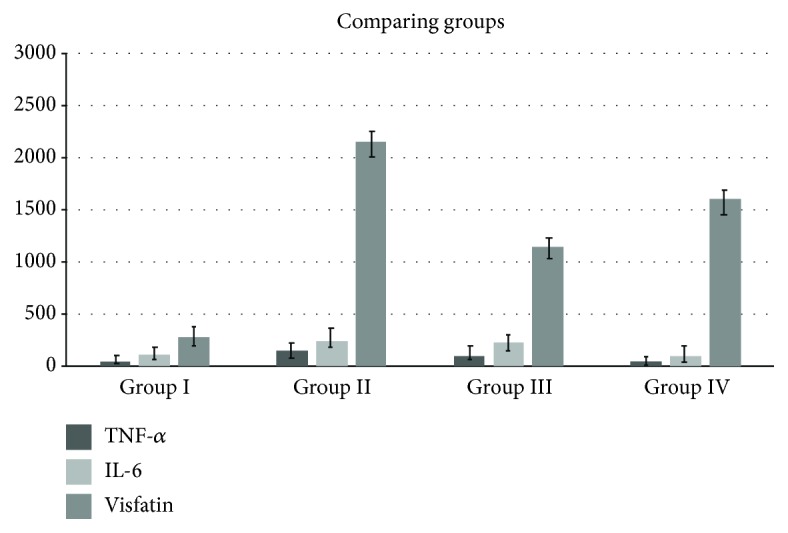
Comparing tumor necrosis factor-*α*, interleukin-6, and visfatin levels among different groups.

Table 1 has illustrated the results of the descriptive analysis for GCF. With respect to IL-6, TNF-*α*, and visfatin, every sample within every group showed positive testing. Table 1 and Figure 1 have additionally demonstrated the average levels of IL-6, TNF-*α*, and visfatin present within the GCF samples. In group 4, IL-6 and TNF-*α* are low but vistafin remains relatively high because the group 4 patients have been suffering from type 2 diabetes and chronic periodontitis, who had received 20 mg simvastatin as a lipid-lowering agent for 5–10 years.

The levels of visfatin in GCFs of patients in groups 1–4 were 284 ± 157.5, 2463 ± 865.3, 1144.2 ± 2.0, and 1608.6 ± 286.8, respectively. A statistical significance was noted with respect to the variation in levels of visfatin between the participant categories. The patients in group 2 had the highest mean level of TNF-*α* in GCF (158.8 ± 26). However, statistical significance was not noted with respect to the variation in the average TNF-*α* levels between group 4 participants (44 ± 12) and group 1 participants (46.20 ± 12.9). Significant differences in the levels of IL-6 were revealed by statistical analysis, between group 2 participants (251.6 ± 60) and group 1 participants (113 ± 27). Additionally, group 3 participants and group 1 participants also showed variation in this context (226.9 ± 62.4). Nevertheless, no statistically significant variation was observed with respect to the levels of IL-6 between groups 4 and 1 or between groups 3 and 2.

### 3.2. Clinical and Radiographic Results

**Figure 2:**
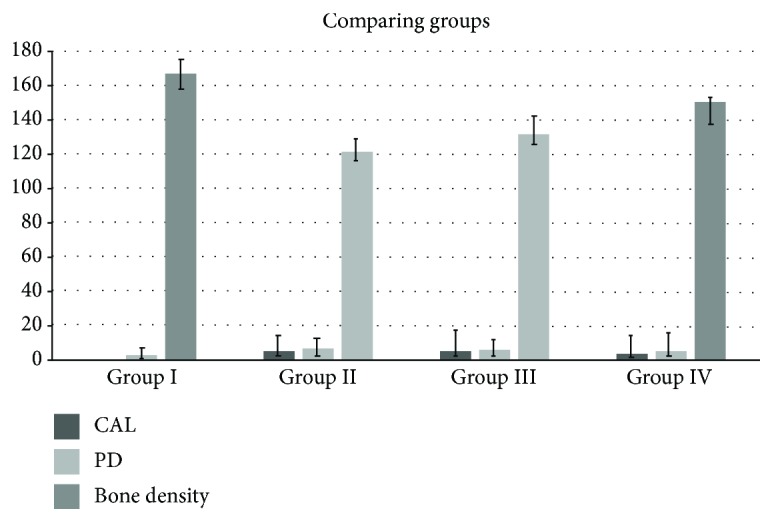
Comparing probing depth, clinical attachment level, and bone density among different groups.

Table 2 and Figure 2 have illustrated the radiographic and clinical variables for each group with respect to the results of descriptive statistics. The clinical parameters pertaining to one site per each participant were carefully analyzed. Radiographic measurements of bone density were recorded for the interproximal alveolar bone at the selected sites.

The variation in parameters of CAL and average probing depth (PD) demonstrated statistical significance with respect to group 1 participants and those belonging to the other groups. It was nevertheless seen that the variation in average CAL and PD values between groups 3 and 2 were not significant. The patients in group 2 had the highest PD (6.5 ± 0.5) and CAL (5.5 ± 0.52) values.

The differences in bone density among the patients in group 1 (167 ± 22.8), group 2 (121.47 ± 22.51), and group 3 (131.67 ± 22.93) were statistically significant; however, those between the patients in groups 2 and 3 were not.

Table 3 has indicated the association between the clinical variables of interest and the GCF levels of IL-6, TNF-*α*, and visfatin. There is a statistically significant and positive association between variables of bone density, CAL, and PD and IL-6, TNF-*α*, and visfatin levels (*P* < 0.001).

## 4. Discussion

The results demonstrated a clear presence of IL-6, TNF-*α*, and visfatin in each GCF sample. Specifically, it was seen that patients belonging to group 2 showed significantly higher levels of IL-6, TNF-*α*, and visfatin as compared to patients from groups 1 and 3. Group 2 was composed of patients that suffered from both diabetes and chronic periodontitis. These results were further corroborated by additional research, which observed increasing levels of visfatin among diabetic patients afflicted with chronic periodontitis [6]. In fact, a positive relationship has been demonstrated between chronic periodontitis and the concentration of visfatin within GCF [7, 8].

Additionally, the results of the present study demonstrated low levels of IL-6 and TNF-*α* among patients belonging to group 4; however, visfatin levels were still seen to be high. As discussed earlier, group 4 consisted of diabetic patients afflicted with chronic periodontitis who had undergone simvastatin treatment. Previous studies had remarked upon the reaction of visfatin with inflammatory cytokines such as TNF-*α* and IL-6 to induce the development of proinflammatory artherosclerosis [9]. Simvastatin treatment has been shown to greatly modulate the cascade mechanisms of inflammatory cytokines [10], through appropriate suppression of cytokine expression [11]. This was clearly represented through the lack of difference in IL-6 levels among patients with healthy periodontitis (group 1) and diabetic patients afflicted with chronic periodontitis who were undergoing simvastatin treatment (group 4). Statins such as simvastatin have been shown to successfully treat osteoporosis, through selective action on bone tissue and inhibition of osteoclast action. Additionally, statins have been seen to have pleitropic impacts such as anti-inflammation and immunomodulation [12].

The anti-inflammatory impact of simvastatin is highlighted through its ability to decrease the production of significant isoprenoid intermediates, through which the production of L-mevalonic acid is inhibited. Such isoprenoid intermediates are inclusive of geranylgeranylpyrophosphate (GGPP) and farnesylpyrophosphate (FPP), which are part of the cholesterol biosynthetic pathway. These isoprenoid intermediates are discussed to be extremely significant in the prenylation of proteins involved in various cellular functions [13]. Furthermore, simvastatin was seen to play a positive role in the promotion of downstream pathway activity, through which, the expression levels of inflammatory cytokines were greatly reduced [14]. The anti-inflammatory applications of simvastatin make it a promising candidate to be used for patients afflicted with periodontitis. With respect to chronic periodontitis, a strong link was seen between simvastatin treatment and the reduction of systemic health parameters such as endothelial dysfunction and inflammation [15]. The study further noted that alveolar bone loss was greatly decreased through simvastatin usage, in addition to enhancing the blood lipic profile, which are all important systemic parameters implicated in chronic periodontitis. However, it was conversely noted that the use of statins was associated with increasing the risk of diabetes mellitus [16]. Nevertheless, this risk is considerably insignificant as compared to the advantages posed through the use of statins.

The results of the present study noted an insignificant difference in IL-6 levels among patients afflicted with both diabetes and periodontitis (group 2) and those patients who suffered only from chronic periodontitis (group 3). This is indicative of the key association between chronic periodontitis and higher levels of visfatin, IL-6, and TNF-*α*. In fact, the role of visfatin as a potential marker for periodontitis had previously been highlighted [17]. Locally delivered simvastatin was seen to play a positive role in chronic periodontitis treatment and impacts IL-6 mRNA levels [18]. Through this, hard and clinical tissue variables were seen to be significantly enhanced.

It was additionally seen that group 2 patients demonstrated the highest values pertaining to the clinical parameters of PD and CAL, as compared to the other three groups. As highlighted previously, this group was the same that had demonstrated the highest visfatin, IL-6, and TNF-*α* levels as compared to the other groups. Therefore, it may be seen that there is a close association between inflammatory cytokines and clinical parameters such as the ones discussed. For instance, a previous study has established the presence of a key link between inflammatory cytokines and the deterioration of systemic health [19]. The same study discussed that IL-6 was associated with greater PD in cardiovascular patients suffering from chronic periodontitis and highlighted its role in inducing the synthesis of c-reactive proteins in cardiovascular patients. There is a limited number of studies that directly address the role of inflammatory cytokines in diabetic patients afflicted with chronic periodontitis; the present study is therefore novel in this context. However, previous research has discussed the role of IL-6 and TNF-*α* in suppressing the insulin signalling cascade, thereby causing a greater resistance to insulin [20]. The exacerbation of systemic inflammation due to proinflammatory cytokines has a detrimental effect on systemic health [21].

It was seen that variations in bone density were significant between group 1, 2 and 3 patients, but variations between groups 2 and 3 were not. Specifically, patients belonging to group 1 showed the highest bone density and those belonging to group 2 showed the lowest. In fact, periodontitis has been shown to have a detrimental impact on bone density, as evidenced by previous literature. Insignificant difference was noted between patients with a healthy periodontium and those who had undergone simvastatin treatment. This is indicative of the positive role of statins such as that of impacting bone density.

## 5. Conclusion

The results of the study were demonstrative of increased IL-6, TNF-*α*, and visfatin levels in diabetic patients afflicted with periodontitis. Moreover, simvastatin may reduce the production of these cytokines, which could make its use favorable in improving periodontal health among diabetic patients. The risks and comorbidities of cardiovascular diseases and diabetes, among other systemic illnesses could be greatly reduced through simvastatin treatment. It is because the results indicated that simvastatin can reduce the inflammatory burden in systemically compromised individuals. Nevertheless, these results could be enhanced through further longitudinal studies that recruit greater sample populations. Such studies pose a great potential in providing useful knowledge pertaining to the management of diabetic patients afflicted with chronic periodontitis. Moreover, it is very important to educate diabetic patients about the importance of lifestyle changes and meticulous home care that can minimize destruction of the periodontium. In the present study, it was extremely difficult to identify a clear relationship between the effect of the drug of interest (simvastatin) and the level of inflammation as it is a cross-sectional study.

## Figures and Tables

**Table 1:** Comparison of groups by tumor necrosis factor-*α*, interleukin-6, and visfatin levels in gingival crevicular fluid.

	Group 1	Group 2	Group 3	Group 4	One-way ANOVA test		Post hoc analysis					
					*F*	*P* value	P1	P2	P3	P4	P5	P6
TNF-*α*	46.20 ± 12.9	158.8 ± 26	98.9 ± 51.5	44 ± 12	32.285	0.001	0.000	0.006	0.698	0.004	0.000	0.004
IL-6	113 ± 27	251.6 ± 60	226.9 ± 62.4	104 ± 46.2	22.453	0.001	0.000	0.000	0.601	0.379	0.000	0.000
Visfatin	284 ± 157.5	2463 ± 865.3	1144.2 ± 2.0	1608.6 ± 286.8	38.667	0.001	0.000	0.000	0.000	0.000	0.008	0.001

ANOVA, analysis of variance; TNF-*α*, tumor necrosis factor-*α*; IL-6, interleukin-6. The data are presented as mean ± standard deviation. *P* < 0.05 is statistically significant. P1: comparison between groups 1 and 2; P2: comparison between groups 1 and 3; P3: comparison between groups 1 and 4; P4: comparison between groups 2 and 3; P5: Comparison between groups 2 and 4; P6: comparison between groups 3 and 4.

**Table 2:** Comparison of groups by probing depth, clinical attachment level, and bone density.

	Group I	Group II	Group III	Group IV	One-way ANOVA test		Post hoc analysis					
					*F*	*P* value	P1	P2	P3	P4	P5	P6
CAL	0 ± 0	5.5 ± 0.52	5.3 ± 0.95	3.9 ± 0.87	134.919	0.001	0.001	0.000	0.000	0.567	0.000	0.003
PD	2.6 ± 0.38	6.5 ± 0.5	5.7 ± 1.76	5.6 ± 0.84	28.016	0.001	0.000	0.001	0.000	0.184	0.009	0.873
Bone density	167 ± 22.8	121.47 ± 22.51	131.67 ± 22.93	150.64 ± 22.5	7.940	0.001	0.001	0.003	0.124	0.329	0.010	0.078

CAL, clinical attachment level; PD, probing depth. The data are presented as mean ± standard deviation. *P* < 0.05 is statistically significant. P1: comparison between groups 1 and 2; P2: comparison between groups 1 and 3; P3: comparison between groups 1 and 4; P4: comparison between groups 2 and 3; P5: comparison between groups 2 and 4; P6: comparison between groups 3 and 4.

**Table 3:** Correlations between the levels of visfatin, tumor necrosis factor-*α*, and interleukin-6 in gingival crevicular fluid and probing depth, clinical attachment level, and bone density.

	TNF-*α*		IL-6		Visfatin	
	*R*	*P* value	*R*	*P* value	*R*	*P* value
CAL	0.728	0.001	0.727	0.001	0.766	0.001
PD	0.622	0.001	0.620	0.001	0.590	0.001
Bone density	0.641	0.001	0.642	0.001	0.568	0.002

*r*, Spearman's rank correlation coefficient; CAL, clinical attachment level; PD, probing depth; TNF-*α*, tumor necrosis factor-*α*; IL-6, interleukin-6. *P* < 0.05 is statistically significant.

## Data Availability

The data used to support the findings of this study are available from the corresponding author upon request.

## Abbreviations

TNF*α*: Tumor necrosis factor alpha
IL: Interleukin
GCF/s: Gingival crevicular fluid/s
DM: Diabetes mellitus
CAL: Clinical attachment level
PD: Probing depth
DSR: Deanship of scientific research.
